# Autophagy May Allow a Cell to Forbear Pyroptosis When Confronted With Cytosol-Invasive Bacteria

**DOI:** 10.3389/fimmu.2022.871190

**Published:** 2022-03-29

**Authors:** Carissa K. Harvest, Edward A. Miao

**Affiliations:** ^1^Department of Microbiology and Immunology, University of North Carolina at Chapel Hill, Chapel Hill, NC, United States; ^2^Center for Gastrointestinal Biology and Disease, University of North Carolina at Chapel Hill, Chapel Hill, NC, United States; ^3^Department of Immunology, Duke University, Durham, NC, United States; ^4^Department of Molecular Genetic and Microbiology, Duke University, Durham, NC, United States

**Keywords:** caspase-11, caspase-1, pyroptosis, autophagy, *Burkholderia*, *Shigella*

## Abstract

Inflammatory caspases detect cytosol-invasive Gram-negative bacteria by monitoring for the presence of LPS in the cytosol. This should provide defense against the cytosol-invasive *Burkholderia* and *Shigella* species by lysing the infected cell *via* pyroptosis. However, recent evidence has shown caspase-11 and gasdermin D activation can result in two different outcomes: pyroptosis and autophagy. *Burkholderia cepacia* complex has the ability invade the cytosol but is unable to inhibit caspase-11 and gasdermin D. Yet instead of activating pyroptosis during infection with these bacteria, the autophagy pathway is stimulated through caspases and gasdermin D. In contrast, *Burkholderia thailandensis* can invade the cytosol where caspasae-11 and gasdermin D is activated but the result is pyroptosis of the infected cell. In this review we propose a hypothetical model to explain why autophagy would be the solution to kill one type of *Burkholderia* species, but another *Burkholderia* species is killed by pyroptosis. For pathogens with high virulence, pyroptosis is the only solution to kill bacteria. This explains why some pathogens, such as *Shigella* have evolved methods to inhibit caspase-11 and gasdermin D as well as autophagy. We also discuss similar regulatory steps that affect caspase-1 that may permit the cell to forbear undergoing pyroptosis after caspase-1 activates in response to bacteria with partially effective virulence factors.

## Introduction

During infection, intracellular pathogens can be recognized through inflammasome sensor pathways that activate caspase-1 or caspase-11, the details of which are extensively reviewed (1). In this review we will discuss the detection of the bacterial type III secretion system (T3SS) as well as cytosolic LPS. Bacterial T3SSs allow bacteria to inject virulence effector proteins into the host cytosol, enabling diverse manipulation of the host cell, including cytosolic invasion (2). T3SS activity is detected by the NAIP/NLRC4 inflammasome, which together oligomerize and activate caspase-1. Caspase-1 then activates IL-1β, IL-18, and gasdermin D. Activated gasdermin D oligomerizes and inserts pores in the plasma membrane, allowing extracellular fluid to rush into the cell, causing cell swelling and lysis that is called pyroptosis (3).

Caspase-11 (or its human equivalents capsase-4 and -5) detects cytosol-invasive bacteria by detecting LPS in the cytosol (4, 5). Caspase-11 obligately requires IFN-γ priming, because IFN-γ drives expression of guanylate binding proteins (GBPs). GBPs are IFN-induced GTPases that deposit on the surface of cytosolic bacteria or upon pathogen-containing vacuoles. The functions of GBPs are complex and reviewed elsewhere (6). Briefly, GPBs exert surfactant like properties, which can expose LPS for detection by caspase-11 (7). Once LPS is exposed, caspase-11 deposits upon the surface of bacterial cells (7–10). Like caspase-1, caspase-11 can activate gasdermin D, which leads to pyroptosis, but unlike caspase-1, caspase-11 does not activate IL-1β and IL-18 (11).

The primary purpose of pyroptosis is likely to deprive an intracellular pathogen of a replicative niche. After an infected cell dies, a series of events leads to the destruction of the bacteria (12, 13). Decisive and rapid pyroptotic signaling therefore appears to be a perfect solution to counteract the virulence strategy of intracellular pathogens. However, upon deeper consideration several aspects of pyroptosis would seem to necessitate prudent, rather than decisive, signaling. First, pyroptosis is irreversible and sacrifices the viability of the cell, which could be a significant cost to the host. Second, pyroptosis may be the best immunologic response in some cell types, but not in others. Finally, the sensors that detect bacterial virulence might have an appreciable rate of false positive activation. These caveats could be especially important since pathogens exist on a spectrum of virulence capacity. Some pathogens are highly virulent, whereas others may have only partially effective virulence factors. In this review, we propose a hypothesis that evolution has selected for regulating checkpoints of pyroptosis, enabling the use of high sensitivity detectors while avoiding inappropriate pyroptosis.

## *Burkholderia thailandensis* and Pyroptosis

*Burkholderia thailandensis* is an environmental pathogen that has immense virulence potential; however, this potential is fully counteracted by caspase-11 (14, 15). *B. thailandensis* encodes a T3SS (which is closely related to the *Shigella* T3SS) that enables cytosolic invasion (16). However, *B. thailandensis* infections in humans are exceedingly rare, and mice are highly resistant to infection (17). Wild type mice clear infections by *B. thailandensis* with incredible efficiency, sterilizing a systemic challenge with 20,000,000 bacterial cells within 1 day (15, 18). However, both *Casp11*^–/–^ and *Gsdmd*^–/–^ mice are susceptible to as few as 100 bacteria (15, 18). This susceptibility is primarily driven by replication of the bacteria within neutrophils (18), resulting in an estimated >1,000,000 fold change in the lethal dose (1), leading us to classify *B. thailandensis* as a high virulence pathogen, but only in the absence of inflammasomes. In summary, *B. thailandensis* cannot evade caspase-11-dependent pyroptosis, explaining why humans and mice are not natural hosts for *B. thailandensis*. The natural host infected by *B. thailandensis* is unknown; we speculate that this host lacks the caspase-11-dependent pyroptosis pathway.

The extremely efficient defense against *B. thailandensis* conferred by caspase-11 and gasdermin D illustrate the importance of accomplishing pyroptosis to counteract cytosolic invasion. If we consider only this class of highly virulent pathogens, what might be the optimal design of the caspase-11 signaling pathway? In such a case, caspase-11 should be highly sensitive, and should cause pyroptosis as quickly as possible. There would be no need to slow down the signaling process because the host cell must be driven to pyroptosis as fast as possible. Thus, *B. thailandensis* provides a very simplistic and effective example of how caspase-11 drives pyroptosis to clear an infection.

However, *B. thailandensis* also simultaneously illustrates the complexities of pyroptotic signaling between caspase-1 and caspase-11. This complexity arises because different cell types have distinct outcomes after inflammatory caspases are activated. *Casp11*^–/–^ mice still have an intact NLRC4 pathway to caspase-1 that should lead to pyroptosis. However, the predicted redundancy that should exist between caspase-1 and caspase-11 is not observed *in vivo* (15). By unknown mechanisms, NLRC4 activation in neutrophils successfully detects the *B. thailandensis* T3SS but fails to cause pyroptosis (18). Why has evolution created a system where NLRC4 does not cause pyroptosis in neutrophils but does so in a macrophage? This likely reflects a flaw in the NLRC4 detection mechanism that is only important for neutrophils – and how evolution has corrected for that flaw, meanwhile maintaining neutrophil defenses against cytosolic-invasion by using caspase-11. The flaw of NLRC4, we speculate, is that it cannot discriminate between a T3SS that is optimized to commandeer a neutrophil from a T3SS that is optimized to commandeer an epithelial cell. If a neutrophil encounters a bacterium with a neutrophil-optimized T3SS, the neutrophil could become infected and harbor replicating bacteria. However, if a neutrophil encounters a bacterium with an epithelial cell-optimized T3SS, the neutrophil will likely phagocytose and kill the bacterium. Thus, if NLRC4 were obligately tied to pyroptosis in neutrophils, the immune system would often inappropriately sacrifice its most lethal attackers. In contrast, we speculate that macrophages are much more dispensable in defense. If a macrophage is intoxicated with an epithelial optimized T3SS, loss of this macrophage is acceptable because neutrophils will arrive soon to continue the fight. This is an example of how pyroptotic signaling might need to use distinct signaling mechanisms in different cell types.

## *Burkholderia cepacia* Complex and Autophagy

The *Burkholderia* cepacia complex (BCC) is a group of opportunistic environmental pathogens, including *Burkholderia cepacia* and *Burkholderia cenocepacia*, that only infect patients with immunocompromising conditions such as cystic fibrosis and chronic granulomatous disease (19, 20). Whereas *B. thailandensis* uses its T3SS to efficiently invade the cytosol, most other *Burkholderia* species lack this *Shigella-*like T3SS. However, many BCC encode a different T3SS (21) that is more closely related to T3SS genes found in *Ralstonia*, a plant pathogen (BLAST results using BscN; GenBank: CAR55907.1). This T3SS may contribute to murine infection (21), but further study is warranted.

BCC encodes other virulence factors such as a T6SS, which can mediate escape from the phagosome in macrophages (22). The T6SS effector TecA is detected by the pyrin inflammasome. This results in a >2 fold change in the lethal dose during high dose infection (10^8^ CFU) in pyrin knockout mice (23), although no phenotype was seen in *Gsdmd*^–/–^ mice using slightly different infection conditions (24). BCC has a much stronger phenotype in NADPH oxidase deficient mice that model chronic granulomatous disease. In these mice, even low dose 10^3^ CFU challenges are lethal (25), demonstrating the significant virulence potential of the BCC. This leads us to define the BCC as intermediate virulence pathogens in the absence of inflammasome defenses.

BCC are also detected by caspase-11 (24, 26), indicating the BCC LPS has entered the cytosolic compartment and supporting the escape of BCC from the vacuole. Although pyroptosis occurs in BCC infected cells, the autophagy pathway is also activated. Remarkably, this autophagy activation requires both caspase-11 and gasdermin D. Furthermore, autophagy restricts BCC replication in macrophages (27, 28). Autophagy is the process that forms a double membrane around unwanted cellular components or invasive bacteria; these autophagosomes are then fused with the lysosome for degradation of their contents (29). The proposed mechanism whereby gasdermin D stimulates autophagy is through pore insertion into mitochondrial membranes, promoting the production of mitochondrial reactive oxygen species (ROS) (24). Autophagy can then kill bacteria. Defective mitochondria are well established to stimulate autophagy (29), but whether there are mechanisms that can specifically target autophagosomes to BCC as opposed to a cell-wide autophagy response remains to be explored. In summary, caspase-11 and gasdermin D can stimulate autophagy.

**Figure 1:**
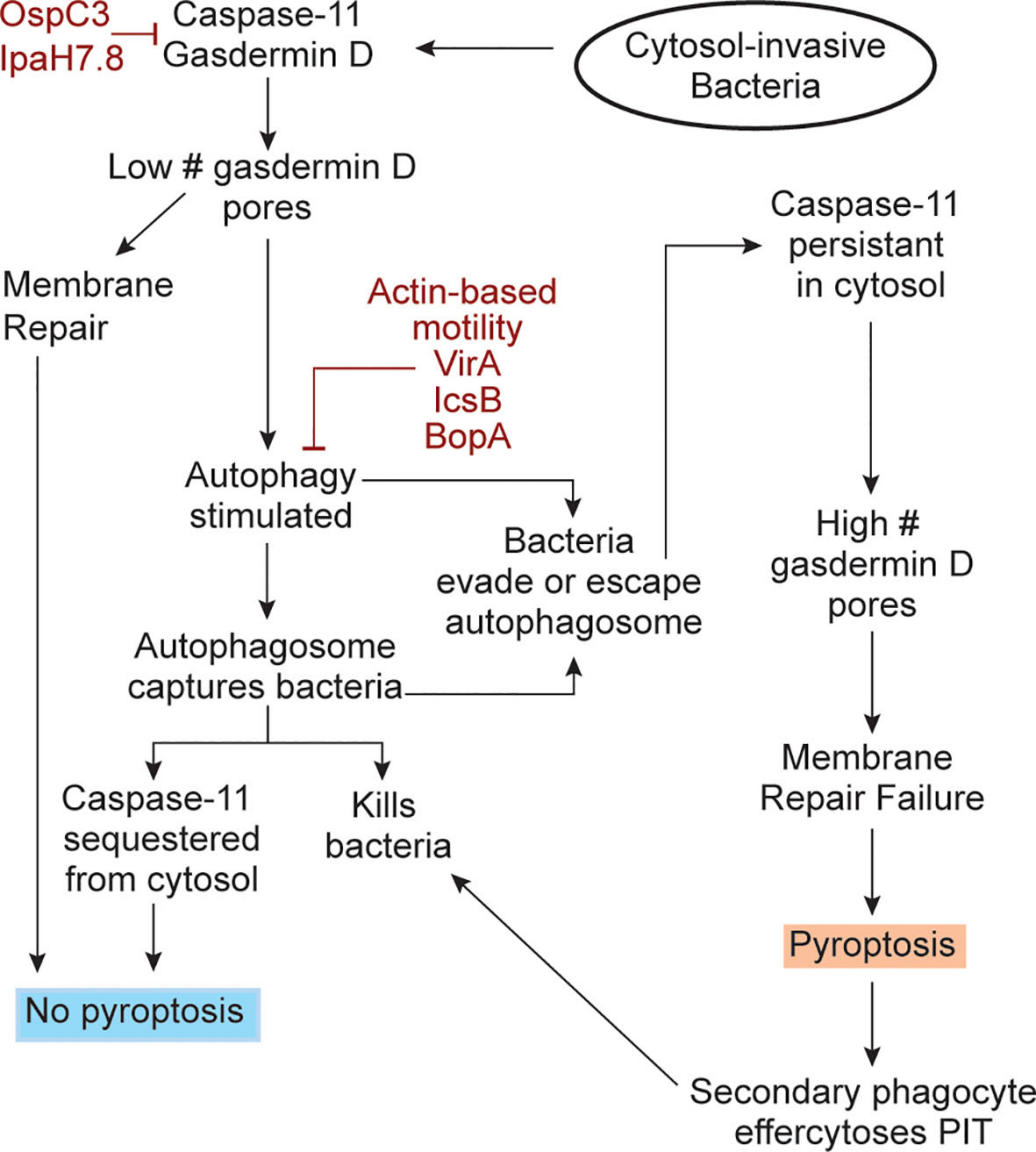
An algorithmic response to cytosol-invasive bacteria. Upon bacterial invasion into the cytosol, caspase-11 is activated by bacterial LPS. Caspase-11 then activates gasdermin D and both simulate the autophagy response. Gasdermin D inserts pores into nearby organelles, such as the mitochondria. The resulting ROS release into the cytosol stimulates autophagy, which captures cytosolic bacteria. There are two significant consequences of this. First, the bacteria can now be killed by the autophagosome. Second, caspase-11 should be simultaneously sequestered from the cytosol and if so, caspase-11 will no longer cleave gasdermin D, thereby preventing pyroptosis. Sequestering of caspase-11 should terminate the gasdermin D cleavage process, and the already generated low amount of gasdermin D pores can be removed from the plasma membrane by membrane repair. If bacterial use virulence factors evade or escape autophagic defenses (burgundy), caspase-11 activity should persist in the cytosol and copious quantities of gasdermin D are expected to be activated. These gasdermin D pores will now insert into the plasma membrane in sufficient quantities and cause pyroptosis. After pyroptosis, the dead cell becomes a pore-induced intracellular trap (PIT) that restrains the bacterium. Secondary phagocytes will be recruited to efferocytose the PITs and the bacteria trapped within it, therefore killing the bacteria. The bacterial virulence factors that inhibit caspase-11, gasdermin D, and the autophagy pathway are shown (burgundy color).

Upon superficial consideration, autophagy and pyroptosis seem counter-functional to each other because a pyroptotic cell loses all metabolism and thus could not perform autophagy. However, upon deeper consideration we propose a hypothetical model wherein autophagy and pyroptosis are not counter-functional. From an overarching perspective, both work to solve the problem of cytosolic replication, albeit through different strategies. Autophagy solves the problem of intracellular infection immediately within the infected cell by killing the bacteria. Pyroptosis solves the problem of intracellular infection by recruiting secondary phagocytes to efferocytose the PIT trapped bacteria. For autophagy and pyroptosis to work together, we speculate that they must occur in an algorithmic manner (**Figure 1**), where an infected cell that activates caspase-11 first attempts autophagy, and only if this fails to eliminate the bacteria does the cell undergo pyroptosis. In this hypothetical model, the first step of the algorithm is for caspase-11 cleavage of gasdermin-D to stimulate autophagy. The BCC is now sequestered from the cytosol and can be killed by the autophagosome. Additionally, because caspase-11 is known to deposit directly to the bacterial outer membrane upon its activation and remain localized there (7–10), we intuit that autophagy of the bacterium will sequester caspase-11 within the autophagosome. This should halt its cleavage of additional gasdermin D molecules, and thereby prevent pyroptosis. If a few gasdermin D pores do reach the plasma membrane, they should be low enough in numbers that basal membrane repair can remove the pores before the cell lyses (30–32).

Under this logic, pyroptosis is only halted if autophagy successfully keeps the bacterium out of the cytosol. If autophagy fails to enclose the bacterium, or if the bacterium escapes from the autophagosome, then the bacterium is again in the cytosol. We propose that this is what happens during *B. thailandensis* infection, perhaps mediated by actin-based motility, which is known to enable evasion of autophagosomes in related pathogens (33). *B. thailandensis* also encodes the effector BopA, which has been shown to inhibit autophagy in other *Burkholderia* species (34). In this case, caspase-11 will again be present in the cytosol and will cleave more and more gasdermin D. The increasing number of pores should overwhelm the membrane repair capacity of the cell and thus cause pyroptosis. This speculative model (**Figure 1**) allows the host cell to perform an algorithm after caspase-11 activation, using autophagy against an intermediate virulence bacterium that invades the cytosol (e.g. BCC), and when this fails, to use pyroptosis against a high virulence bacterium (e.g. *B. thailandensis*).

## Autophagy and Self-Regulation of Caspase-1

**Figure 2:**
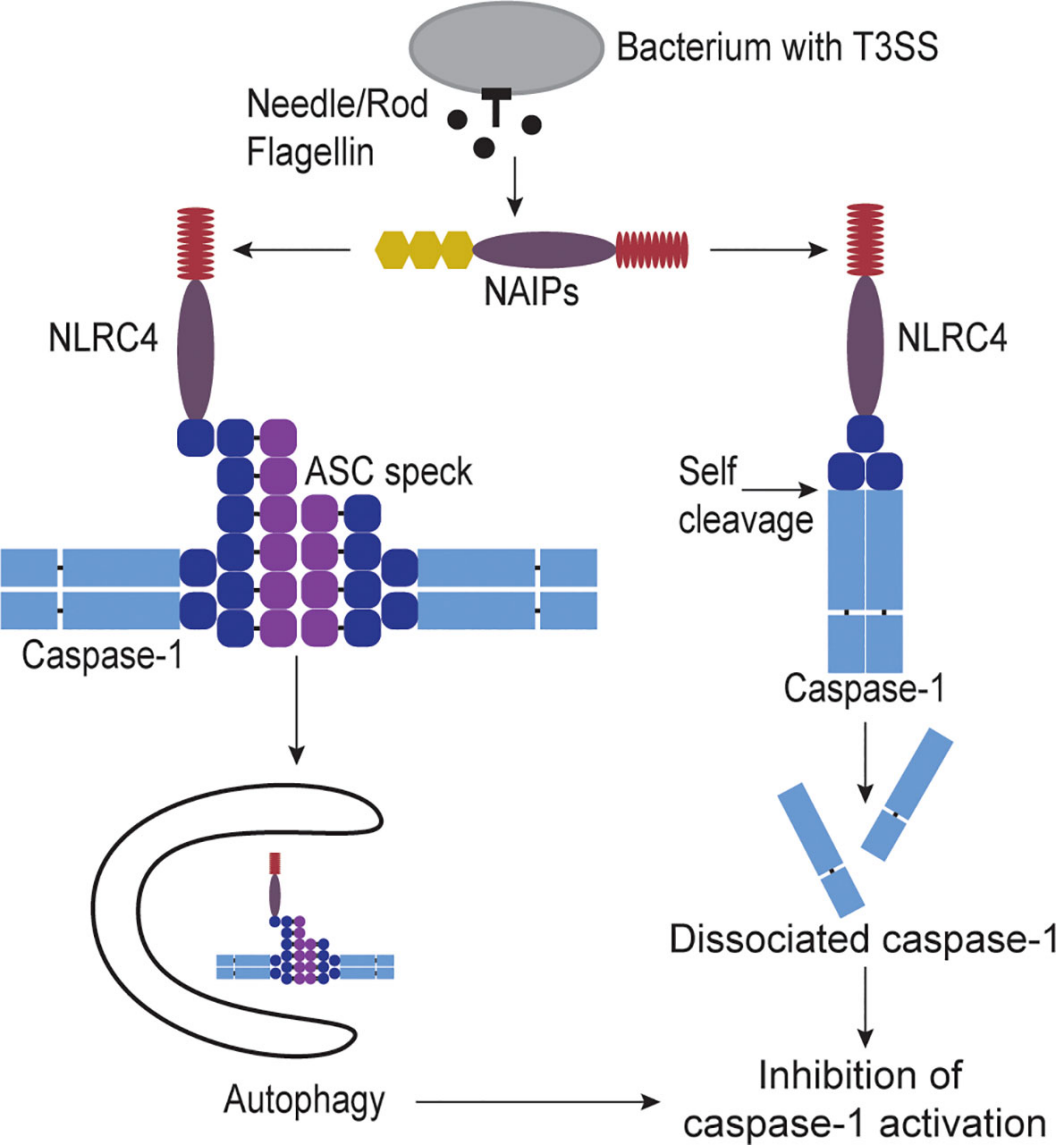
Regulation of caspase-1 activity. When cytosolic invasive bacteria accidently secrete flagellin, T3SS rod, or T3SS needle proteins they are detected by NAIPs. NAIPs activate NLRC4 which in turn recruits and activates caspase-1. Once caspase-1 is activated it can activate gasdermin D to form pores in the plasma membrane causing cell swelling and lysis called pyroptosis. However, caspase-1 has two mechanisms for inactivation. One way to inhibit caspase-1 is through sequestration of caspase-1 from the cytosol by capturing it in autophagosomes. NLRC4 has been shown to activate the autophagy pathway. NLRC4 also can recruit the adaptor protein ASC speck which can bind multiple capase-1 proteins. This ASC speck is large enough that the autophagy pathway can recognize and engulf the protein complex, therefore capturing the bound caspase-1 as well. Another way to inhibit caspase-1 is through self-cleavage by caspase-1 itself. NLRC4 or ASC directly bind caspase-1, bringing the proteins close enough to dimerize. Caspase-1 can then directly cleave between its CARD and protease domains. This cleavage releases the protease from the activating platform, allowing the caspase protease domains to disassociate, rendering caspase-1 inactive.

The autophagic mechanisms discussed above to inactivate caspase-11 may also apply to caspase-1, albeit in a modified form. First, when the NLRC4 inflammasome activates caspase-1, this has also been shown to stimulate autophagy (35), presumably through the same mechanism where gasdermin D pores insert into mitochondria. However, the mechanism of autophagy sequestering caspase-1 must be different from caspase-11 because caspase-1 does not deposit upon bacteria. In this regard, caspase-1 activation often involves an adaptor protein, ASC, that maintains a specific localization of the active caspase (36). After inflammasomes activate, they recruit ASC, which then polymerizes to form the ASC speck. The speck directly binds caspase-1, which remains attached to the ASC speck. This ASC speck can be autophagocytosed (**Figure 2**), resulting in inhibition of caspase-1 activity (37). Autophagy can also regulate other inflammasomes at earlier steps, notably in the activation step of NLRP3 (38).

Caspase-1 also has an additional method to halt its activity, which is by self-inactivating cleavage (39). Caspase-1 can cleave itself between its CARD and protease domains. As the CARD domain is essential to maintain a dimerized state, the loss of the CARD results in disassociation of the protease domain (**Figure 2**), thereby inactivating caspase-1 (39). Notably, this cleavage event should also release the caspase-1 protease domain from the ASC speck, thus, caspase-1 should only escapes autophagy in an inactive form. In summary, capsase-1 simultaneously has a robust amplification step in polymerization of the ASC speck, but this is paired with greater complexity in opportunities for inactivation. Notably, both the ASC amplification and the CARD-protease cleavage mechanisms are absent in caspase-11.

## *Shigella* and Inhibition of Pyroptosis

Notably, *B. thailandensis* and BCC only cause human infection in immunocompromised people (17, 19). This illustrates the potent defense conferred by pyroptosis and autophagy against pathogens that have intermediate or even high virulence potential, but which lack the ability to inhibit these defenses. *Shigella* is a pathogen with high virulence that additionally can inhibit these defenses (40, 41). *Shigella dysenteriae* and *Shigella flexneri* cause the disease Shigellosis, which is characterized by hemorrhagic diarrhea and is particularly dangerous to young children (42, 43). These pathogens are highly infectious requiring ingestion of only 10-100 bacteria to cause disease (9, 44–46). Both *S. dysenteriae* and *S. flexneri* encode a T3SS that orchestrates cytosolic invasion, which is closely related to the T3SS used by *B. thailandensis* (16).

Caspase-11 should detect the *Shigella* LPS when the bacterium invades the cytosol, and the resulting pyroptosis would eliminate the infected cell niche. However, *Shigella* uses the T3SS effector OspC3 to inhibit caspase-11 (46). OspC3 uses a unique biochemical reaction to catalyze the ADP-riboxanation of a critical arginine residue of caspase-11, which prevents activation of the caspase protease domain (46). Nevertheless, the innate immune system should still detect the *Shigella* T3SS *via* NAIP/NLRC4, activating caspase-1 and gasdermin D. However, *Shigella* can also inhibit gasdermin D with the T3SS effector IpaH7.8, which ubiquitinates both gasdermin D and gasdermin B to drive its proteasomal degradation (41, 47). In the evolutionary battle between hosts and pathogens, OspC3 and IpaH7.8 put *Shigella* two steps ahead of cytosolic sensors that would cause pyroptosis and autophagy. Therefore, every aspect of the caspase-1/11 and gasdermin D defense pathway that works against *B. thailandensis* and BCC fails against *Shigella*. Finally, *Shigella* is known to inhibit autophagic responses by the T3SS effectors IcsB and VirA (48, 49), as well as escaping autophagosome formation using actin-based motility (33). This undoubtedly underlies the high virulence of *Shigella* species in healthy immunocompetent people.

*Shigella* is not the only pathogen that successfully replicates in the cytosol despite caspase-11. *Burkholderia mallei* and *Burkholderia pseudomallei* encode a *Shigella*-like T3SS that enables cytosolic invasion, and are close relatives of *B. thailandensis* (17, 50). *B. mallei* is a mammalian adapted pathogen that causes glanders, a highly contagious and deadly disease in horses (and a zoonotic disease of humans). *B. pseudomallei* is an environmental bacterium that causes melioidosis, a significant cause of community acquired sepsis in endemic regions (50). Both *B. mallei* and *B. pseudomallei* have sufficient virulence to be classified as potential biological weapons. *B. pseudomallei*, like *Shigella*, can evade killing by the autophagy pathway (51, 52). These bacteria are highly virulent, and *B. pseudomallei*, like Shigella (and presumably *B. mallei*), can evade the autophagy responses, including *via* its IcsB homolog, BopA (51–53). Therefore, we think that *B. mallei* and *B. pseudomallei* must evade caspase-11, unlike *B. thailandensis*. Indeed, *B. pseudomallei* can suppress interferon gamma signaling (54), which could prevent detection by caspase-11. However, the virulence factors accomplishing this remain unidentified.

## Concluding Remarks

In this review we have considered how inflammatory caspases defend against cytosol-invasive bacteria that have various degrees of virulence. We propose a hypothetical model to explain how caspase-1/11 and gasdermin D could cause pyroptosis, while also being associated with the stimulation of autophagy. We propose that intermediate virulence pathogens such as BCC species can invade the cytosol and are there detected by caspase-11. The resulting gasdermin D pores stimulate a cellular autophagy response that can capture and kill the bacteria, while simultaneously silencing caspase-11 signaling. In contrast, *B. thailandensis* presumably escapes this autophagic response, remaining in the cytosol. The persistent caspase-11 signaling produces numerous gasdermin D pores that become sufficient to overcome membrane repair and cause pyroptosis. The ultimate manifestation of cytosolic invasion is exemplified by *Shigella* species, which invade the cytosol while simultaneously inhibiting caspase-11 and gasdermin D as well as autophagy. Evasion of these innate immune defenses is undoubtedly a key to the intense virulence manifested by *Shigella*.

## Author Contributions

CH and EM wrote the article. All authors contributed to the article and approved the submitted version.

## Funding

This publication is supported by AI133236, AI139304, AR072694, AI136920, AI133236-04S1.

## Conflict of Interest

The authors declare that the research was conducted in the absence of any commercial or financial relationships that could be construed as a potential conflict of interest.

## Publisher’s Note

All claims expressed in this article are solely those of the authors and do not necessarily represent those of their affiliated organizations, or those of the publisher, the editors and the reviewers. Any product that may be evaluated in this article, or claim that may be made by its manufacturer, is not guaranteed or endorsed by the publisher.

## Abbreviations

PAMP, Pathogen associated molecular pattern; LPS, lipopolysaccharide; T3SS, type 3 secretion system; NLR, nod-like receptor; NAIP, NLR family, apoptosis inhibitory proteins; NLRC4, NLR family CARD domain containing 4; GBP, guanylate binding proteins; PIT, pore-induced intracellular traps; ASC, apoptosis-associated speck-like protein; BCC, *Burkholderia* cepacia complex; ROS, reactive oxygen species.
